# Digital rock physics and laboratory considerations on a high-porosity volcanic rock

**DOI:** 10.1038/s41598-020-62741-1

**Published:** 2020-04-03

**Authors:** Laura L. Schepp, Benedikt Ahrens, Martin Balcewicz, Mandy Duda, Mathias Nehler, Maria Osorno, David Uribe, Holger Steeb, Benoit Nigon, Ferdinand Stöckhert, Donald A. Swanson, Mirko Siegert, Marcel Gurris, Erik H. Saenger

**Affiliations:** 1 Fraunhofer IEG, Institution for Energy Infrastructures and Geothermal Systems, Bochum, 44801 Germany; 2 Bochum University of Applied Sciences, Department of Civil and Environmental Engineering, Bochum, 44801 Germany; 3 Bochum University of Applied Sciences, Institute of Mathematics and Informatics, Bochum, 44801 Germany; 4 Ruhr-University Bochum, Institute of Geology, Mineralogy and Geophysics, Bochum, 44801 Germany; 5 University of Stuttgart, Institute of Applied Mechanics (CE), Stuttgart, 70569 Germany; 6 University of Stuttgart, SC SimTech, Stuttgart, 70569 Germany; 7 U.S. Geological Survey, Hawaiian Volcano Observatory, Hilo, Hawaii United States

**Keywords:** Solid Earth sciences, Geophysics, Mechanical properties

## Abstract

Digital rock physics combines microtomographic imaging with advanced numerical simulations of effective material properties. It is used to complement laboratory investigations with the aim to gain a deeper understanding of relevant physical processes related to transport and effective mechanical properties. We apply digital rock physics to reticulite, a natural mineral with a strong analogy to synthetic open-cell foams. We consider reticulite an end-member for high-porosity materials with a high stiffness and brittleness. For this specific material, hydro-mechanical experiments are very difficult to perform. Reticulite is a pyroclastic rock formed during intense Hawaiian fountaining events. The honeycombed network of bubbles is supported by glassy threads and forms a structure with a porosity of more than 80%. Comparing experimental with numerical results and theoretical estimates, we demonstrate the high potential of in situ characterization with respect to the investigation of effective material properties. We show that a digital rock physics workflow, so far applied to conventional rocks, yields reasonable results for high-porosity rocks and can be adopted for fabricated foam-like materials with similar properties. Numerically determined porosities, effective elastic properties, thermal conductivities and permeabilities of reticulite show a fair agreement to experimental results that required exeptionally high experimental efforts.

## Introduction

The fundamental aim of rock physics is to determine, understand and model relations between remotely-sensed geophysical observations and in-situ rock properties. Using high resolution representations of the complex pore geometry^1,2^, digital rock physics (DRP) has rapidly emerged as a potential source of valuable rock property relations and fundamental understanding of pore-scale processes governing these properties^3^. The digital rock physics workflow comprises three steps^4^: imaging, segmentation and numerical simulations. A 3D digital image of the rock sample is produced using a tomography method. By segmentation pore space and mineral phases are separated from the images. The obtained microstructural information is used for numerical simulations of physical transport processes to obtain effective properties, such as permeability and elastic moduli.

The present study focuses on DRP applied to a rock material considered as end-member with respect to porosity and stiffness (and brittleness) of the skeleton, reticulite. Reticulite originates from Island of Hawaii (USA), more specifically the southernmost shield volcano the Kīlauea Volcano. It is one of the most studied and best understood basaltic volcanoes in the world^5^. During its long complex eruptive past (about 1470 CE) there were major changes in summit architecture that likely enabled highly variable explosive magmatic and phreatomagmatic behavior. High fountains produced a deposit of reticulite that encircles the caldera. Reticulite is only produced after the caldera formed or during its final stage of collapse. To date the reticulite and constrain the age of caldera collapse charcoal is used that is produced while the reticulite drapes the caldera and burned vegetation^6^.

Reticulite, sometimes called thread-lace scoria, is a frothy basaltic rock and typically brown in color, in places oxidized pink or red, with clast diameters from sub-centimeter to 15–20 cm^6^. The fragile rock exhibits the lowest average density of any rock worldwide due to its porosity (vesicularity) of up to 98%. The open honeycomb network of bubbles (vesicles) results from the bursting of cell walls during vesiculation. It is speculated that the rise speed of magma determines the intensity of the vesiculation burst and, hence, also the vesicle size distribution systematics^7^.

Reticulite samples were collected during and after a fieldtrip of the SEG-AGU workshop “Rock physics of the upper crust” in July 2016 in Hawaii. The DRP approach including the determination of tortuosity, defined as the ratio of flow-path length to length of the sample^8^, was complemented by microscopic measurements of surface structures and by a comprehensive laboratory characterization of physical properties. We evaluated our results with respect to the individual advantages of laboratory measurements, numerical results and theoretical estimations for materials like reticulite for different requirements on accuracy of results and limitations on the efforts to obtain these results. The body of literature of a detailed and comprehensive rock physical characterization of reticulite is to the best of our knowledge so far rare. Appropriate references to specific aspects are given below.

## Materials and Methods

### Sample material

**Figure 1:**
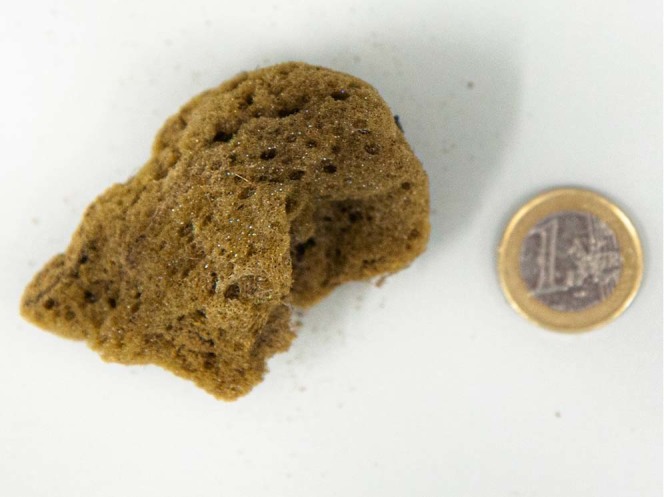
Typical sample of reticulite collected near the Hawaiian Volcano Observatory.

Our sample material originates from a widely dispersed reticulite bed located close to the base of the Keanakāko‘i Tephra at Kīlauea Volcano, Hawaii. An example of a reticulite deposit from location KR08-01, where our set of approximately 30 individual samples (Fig. 1) were collected, is shown in Fig. 16.3 of May *et al*.^6^.

The chemical compositions of most Hawaiian lavas allocate near the silica-poor end and show large amounts of iron, magnesium, and lime. On average, proportions (in wt%) of the principal oxides in the lava of Kilauea were reported to be 48.35% for silicon (SiO${}_{2}$), 13.18% for aluminum (AlO${}_{3}$), 9.2% for magnesium (MgO), and 10.34% for calcium (CaO)^9^. More recent studies by Jackson *et al*. (2012) and Helz *et al*. (2014) confirm these amounts for sample material from the Kīlauea Volcano^10,11^.

Sample preparation and experimental characterization of reticulite was highly challenging. Reticulite shows a spongy appearance, but with a stiff solid frame, and fails on touch. Treatment of stored sample material exposes dust of glassy, spicular fragments with high aspect ratios. We recommend gloves and mouth protection when working with reticulite.

### Microstructure

We characterized the microstructure of reticulite using three different non-destructive testing methods: Digital microscopy, X-Ray computed microtomography (XRCT), and Scanning Electron Microscope (SEM) imaging. The 3D-structure of the solid frame served as input for numerical estimates of effective elastic, thermal and hydraulic properties.

Surface analyses of reticulite were performed using a digital KEYENCE microscope VHX-2000D combined with a VH-Z100UR objective. Surface measurements were achieved using a non-contact optical depth-from-defocus method^12^. This approach provides 3D surface measurements by successive vertical displacement of the distance between object and microscope lens using a digital microscope camera with a small depth of focus and taking images of the object while successively displacing the camera in a vertical direction. The vertical position information is then attributed to the in-focus areas of the respective images. All images were merged into one topographic surface representation with a vertical and an in-plane resolution of less than 10 $\mu$m and 1 $\mu$m, respectively.

X-Ray computed microtomography (XRCT) was used to characterize the 3D pore morphology with $\mu$m-resolution. The procedure allows the direct observation of pore structures in porous media by producing a stack of grey scale images based on absorption, whose grey values to some extent correlates with density, with minimum effort in sample preparation and short scanning time^1,3,13–15^. The investigated 3D datasets were recorded with a ProCon X-Ray system. The XRCT-scanner is equipped with an X-Ray tube of 225 kV voltage and capable of reconstructing 3D details. Scanning of the investigated cubic samples with a side length of approximately 12 mm, 10 mm, and 5 mm took between two and three hours and the reconstruction lasted about one hour. The resolution is controlled by sample position between X-Ray tube and detector. The geometrical magnification $M$ of an X-Ray system is defined by the source-detector distance (SDD) divided by the source-object distance (SOD)^16^. The resulting voxel sizes for the three sample geometries are 16.01 $\mu$m, 12.85 $\mu$m, and 4.48 $\mu$m (Table 1)^17^. Image enhancement and segmentation steps were carried out using the commercial software package Avizo Fire (version Avizo Fire 9.1.1, Thermo-Fisher, FEI Visualization Sciences Group, https://www.thermofisher.com/de/de/home/industrial/electron-microscopy/electron-microscopy-instruments-workflow-solutions/3d-visualization-analysis-software/avizo-materials-science.html). Before actual segmentation the image noise and scan artefacts were reduced while preserving interfaces using a 3D non-local mean denoising filter. Segmented data sets of 400 $\times$ 400 $\times$ 400 voxels can be found in Schepp *et al*.^17^.

**Table 1:** Overview of the selected XRCT-scan parameters for the different samples^17^.

parameter	sample		
	01-AxMF01	03-AxMF01	AxHP-wet
*d* (mm)	10	5	12
$dx$ (${\rm{\mu }}$m)	16.01	4.48	12.85
focal spot	MF	MF	HP
${V}_{{\rm{a}}}$ (kV)	390	440	490
$I$ ($\mu$A)	60	40	90
${t}_{{\rm{e}}}$ (ms)	500	600	350
$SSD$ (mm)	390	440	490
$SOD$ (mm)	50.65	15.90	50.00
filter	1 mm Al	—	1 mm Al

$d$: sample size; $dx$: voxel size; MF: microfocus imaging; HP: high-power imaging; ${V}_{{\rm{a}}}$: acceleration voltage; $I$: electric current; ${t}_{{\rm{e}}}$: exposure duration; SSD: source-detector distance; SOD: source-object distance.

Samples and fragments of the sample were visualized using a high resolution thermally aided field emission Scanning Electron Microscope (SEM) with a resolution of 0.8 nm at 15 kV. The 2D grey-scale images were used to qualitatively specify the structure of the solid frame and the characteristics of the pore connectivity.

### Laboratory measurements

We investigated basic physical properties (matrix density, porosity, ultrasound P- and S-wave velocity) as well as thermal and hydraulic properties of various reticulite samples. In general, individual tests were performed on different samples because of their fragile nature and the requirement of undamaged sample material.

#### Basic physical properties (laboratory)

Matrix density was gained from pycnometer measurements on ground rock powder using distilled water as fluid medium. Cylindrical subsamples or cubes were stamped out of the sample volume for geometric density and ultrasound velocity measurements. End faces of the samples were cut square using a scalpel. Grinding of end faces, as would usually be part of the preparational procedure of rock samples, was not applicable. Total porosity was calculated from the ratio of geometric density to matrix density.

#### Dynamic elastic properties (laboratory)

For ultrasound velocity measurements, two identical ultrasound P-wave transducers (Olympus V153; 100 kHz centre frequency; 1.5 in diameter) acting as source and receiver were placed at both sample ends. Measurements were performed either with or without coupling medium (conventional medical ultrasound gel). An external waveform generator (Panametrics EPOCH 650) produced a rectangular signal with an amplitude of 400 V that activated the piezo-electric source transducer to emit mechanical pulses into the samples. The transmitted elastic waves were stacked and stored using a digital oscilloscope (Picoscope 5444B).

#### Thermal properties (laboratory)

Thermal conductivity $\lambda$ was measured using a thermal conductivity scanner (Lippmann and Rauen GbR; TCS No. 2010-013) according to Popov (1997)^18^. The method is based on scanning a sample surface with a focused, mobile and continuously operating heat source in combination with infrared temperature sensors. Care was taken that samples for thermal conductivity measurements comply with the geometrical requirements on sample dimension to reduce boundary effects.

#### Hydraulic properties (laboratory)

Interconnectivity was tested in untreated samples of reticulite. Classic attempts to determine water permeability in rock samples could not be applied to reticulite samples due to its high sensitivity to any mechanical treatment. The skins connecting the framework were expected to be hydraulically relevant, but any jacketing would have caused the destruction of these skins leading to hydraulic circuits along the jacket-sample interface. Therefore, a simple qualitative test was conducted using a fluid-filled (distilled water or silicon oil) open pipette with an outlet diameter of about 0.5 mm and a maximum fluid volume of 200 $\mu$l . The pipette was carefully stacked into the samples’ centre allowing for a gravitational inflow into the pore space and minimizing hydraulic boundary effects due to the limited sample dimension. Except for the actual entry path of the pipette and its close vicinity, no damage should have been induced by the pipette in the bulk sample volume. The time-dependent fluid level was observed in the transparent top part of the pipette. No fluid leakage was detected at the entry of the pipette ensuring that fluid flow into the sample was dominant rather than along the damaged entry path.

### Numerical counterparts for laboratory measurements

#### Effective elastic properties (numerical)

To obtain effective P- and S-wave velocities of the digitized rock samples we use a technique described in detail in Saenger *et al*. (2004) and references therein^19^. The basic idea of this approach is to study velocities of elastic waves through heterogeneous materials in the long wavelength limit (pore size $\ll$ wavelength). We apply the Rotated Staggered Grid (RSG)-technique to model wave propagation in porous media^20^. The rock-models are embedded in a homogeneous region. Models are defined by $804\times 400\times 400$ grid points with an interval $\delta$ corresponding to that of the XRCT scan. For homogeneous regions we assign a P-wave velocity of ${v}_{{\rm{P}}}\ =5100\ {{\rm{ms}}}^{-1}$, an S-wave velocity of $${v}_{{\rm{S}}}\ =\ $$ 2944 ms${}^{-1}$, and a matrix density of 2540 kg m${}^{-3}$. For dry pores we set ${v}_{{\rm{p}}}$ and ${v}_{{\rm{S}}}$ to 0 ms${}^{-1}$ and ${\rho }_{{\rm{vac}}}$ to 0.0001 kg m${}^{-3}$ approximating vacuum. For water-filled pores we set ${v}_{{\rm{P}}}\,=$ 1500 ms${}^{-1}$, ${v}_{{\rm{S}}}\,=$ 0 ms${}^{-1}$, and ${\rho }_{{\rm{water}}}=1000\ {\rm{k}}{\rm{g}}\ {m}^{-3}$. Schilling *et al*. measured compressional (${v}_{{\rm{P}}}$) and shear wave velocities (${v}_{{\rm{S}}}$) of 20 non-porous glasses in the pseudoternary system anortithe (An) – diopside (Di) – forstertite (Fo)^21^. Their inclusion free sample PO05 with a chemical composition of (among others) 49.52% silicon, 17.48% aluminum, 9.53%, magnesium, and 22.5% calcium is similar to Hawaiian lava and was therefore adopted in terms of their measured values for ${v}_{{\rm{P}}}\,=$ 6697m s${}^{-1}$, $${v}_{{\rm{S}}}\ =\ $$ 3751 m s${}^{-1}$, and a density of ${\rho }_{{\rm{m}}}=$ 2777 kg m${}^{-3}$ to the mineral phase forming the matrix of our reticulite samples from Hawaii.

We perform our numerical modeling with periodic boundary conditions in the two horizontal directions, even though we have no periodic microstructure, because periodic boundary conditions are necessary for a clean generation of plane waves. Andrä *et al*. (2013) show that static and dynamic calculations give very similar results^3^. We apply a body force plane source at the top of the model to obtain effective velocities. The source wavelet in our experiments corresponds to the first derivative of the Gaussian function with a dominant frequency of ${f}_{{\rm{fund}}}=$ (5 ms${}^{-1}$)/$\delta$. The modeled plane P- or S-wave propagates through the porous medium. At two horizontal planes of receivers (top and bottom) we measure the time-delay of the mean peak amplitude of the plane wave caused by the heterogeneous region. With the time-delay one can estimate the effective velocity and the corresponding bulk modulus $K$ and shear modulus ${\rm{\mu }}$. All computations are performed with second order spatial FD operators and with a second order time update.

As described in Saenger *et al*. (2004) our numerical setup enables us to compare effective elastic properties of dry and fluid filled 3D porous media (i.e., the dry rock skeleton is identical in both cases)^19^. We can test the applicability of the Gassmann-equation^22^ and the Biot velocity relations^22–24^ for 3D porous materials without additional effective medium theory. For all synthetic models we fulfill the assumptions of the Gassmann-equation, that is, isotropy, frictionless fluid, undrained system, and no chemical interactions^25^. However, from a theoretical point of view we consider the high frequency range of the Biot velocity relations because we saturate our rock-models with a non-viscous fluid, that is, $${\eta }_{{\rm{fl}}}=0$$. Hence, the reference frequency $${f}_{{\rm{biot}}}$$ can be determined for our rock-models with a non-zero permeability $$k$$ using $${f}_{{\rm{biot}}}=\Phi \eta /(2{\rm{\pi }}{\rho }_{{\rm{fl}}}k)$$ as zero^8^ with porosity $$\phi $$. One geometrical parameter in the Biot velocity relations, the tortuosity parameter $$\tau $$^8^, is challenging to determine analytically. The difference between the high- and the low-frequency limit (i.e. Gassmann equation) of the Biot velocity relations for the fast P- and the S-wave becomes largest for $$\tau $$ = 1 and zero for $$\tau $$$$\to $$$$\infty $$. This can be evaluated for S-waves by analyzing the corresponding prediction of the Biot approach^8^

1 $${v}_{s,\infty }={\left(\frac{{{\rm{\mu }}}_{{\rm{dry}}}}{\rho -\phi {\rho }_{{\rm{fl}}}{\tau }^{-1}}\right)}^{0.5},$$

where ${v}_{{\rm{s}},\infty }$, ${{\rm{\mu }}}_{{\rm{dry}}}$, and ${\rho }_{{\rm{fl}}}$ denote high-frequency limiting shear velocity, effective shear modulus of the dry rock skeleton and fluid density, respectively. The density of the porous material is derived according to $\rho =(1-\phi ){\rho }_{{\rm{grain}}}+\phi {\rho }_{{\rm{fl}}}$. The equation for the velocity of the fast P-wave with the tortuosity-behavior described above is also given by Mavko *et al*.^8^.

For rock samples with moderate porosities (up to 20%) the difference between the high- and the low-frequency limit of the Biot velocity relations is rather low. According to Saenger *et al*. (2004) the rock structure is saturated with an imaginary fluid of high density (${v}_{{\rm{P}}}\,=$ 1500 m s${}^{-1}$; ${v}_{{\rm{S}}}\,=$ 0 m ${s}^{-1}$; ${\rho }_{{\rm{fluid}}}\,=$ 15000 kg m${}^{-3}$)^19^. For such a saturation scenario the difference between low- and high-frequency limit of the Biot-approach increases and the determination of the tortuosity becomes numerically feasible.

#### Effective thermal conductivity (numerical)

In order to numerically determine the effective thermal conductivity of the digitized reticulite samples, we developed a solver based on the cell-centered finite volume method. The starting point is the general energy balance of a 3D infinitesimal control volume according to

2 $$\frac{{\rm{\partial }}(\rho {E}_{{\rm{t}}{\rm{o}}{\rm{t}}})}{{\rm{\partial }}t}+\overrightarrow{{\rm{\nabla }}}\cdot (\rho {E}_{{\rm{t}}{\rm{o}}{\rm{t}}}\overrightarrow{v})=\overrightarrow{{\rm{\nabla }}}\cdot (\overrightarrow{\sigma }\cdot \overrightarrow{v}-{\dot{\overrightarrow{q}}}_{{\rm{c}}})+\rho \overrightarrow{g}\cdot \overrightarrow{v}+{\dot{q}}_{{\rm{r}}{\rm{a}}{\rm{d}}}+{q}_{{\rm{s}}},$$

where ${E}_{{\rm{tot}}}$, $\overrightarrow{v}$, $\overrightarrow{\sigma }$, ${\dot{\overrightarrow{q}}}_{{\rm{c}}}$, $\overrightarrow{g}$, ${\dot{q}}_{{\rm{rad}}}$, and ${q}_{{\rm{s}}}$ denote total energy, velocity vector, Cauchy stress tensor, conductive heat flux vector, gravitational field, radiative heat flux and heat source, respectively. We consider simplified assumptions to reduce the computational complexity of the model:

- The model is assumed to be at steady state, that is, $\partial /\partial t=0$.
- Air is treated as a quasi-solid and thus, convective effects are neglected, that is, $\overrightarrow{v}=\overrightarrow{0}$.
- Heat conduction is modelled with Fourier’s law: ${\dot{\overrightarrow{q}}}_{{\rm{c}}}=-\,\lambda \ \overrightarrow{\nabla }T$.
- We assume that there is no source term that generates heat $({\dot{q}}_{{\rm{s}}}=0)$, that is, the heat-conduction equation is homogeneous.
- Radiation is assumed to have a small impact on the effective thermal conductivity and is hence neglected, that is, ${\dot{q}}_{{\rm{rad}}}=0$.
- Local heat conductivity may vary in space, $\lambda (x,y,z)$, but exhibits no temperature dependence: $\lambda \ne \lambda (T)$.

Using these assumptions the found model equation writes as

3 $$-\overrightarrow{\nabla }\cdot (\lambda \ \overrightarrow{\nabla }T)=0.$$

Discretization of Eq. (3) requires the evaluation of the term $\lambda \ \overrightarrow{\nabla }T$ at cell surfaces (Fig. 10). Whereas the temperature gradient is computed fairly straightforward following the central differencing scheme, the evaluation of $\lambda$ is more demanding. The calculation of $\lambda$ requires heat flux consistency $({\dot{\overrightarrow{q}}}_{{\rm{c,1a}}}={\dot{\overrightarrow{q}}}_{{\rm{c,2b}}})$ and temperature consistency $({T}_{{\rm{a}}}={T}_{{\rm{b}}})$. These conditions lead to the well known harmonic average of Patankar^26^, namely

4 $${\lambda }_{{\rm{f}}}={\left(\frac{1-{f}_{{\rm{e}}}}{{\lambda }_{1}}+\frac{{f}_{{\rm{e}}}}{{\lambda }_{2}}\right)}^{-1}\,{\rm{with}}\,\,{f}_{{\rm{e}}}=\frac{{x}_{2}-{x}_{{\rm{f}}}}{{x}_{2}-{x}_{1}}.$$

For more information about the technical implementation of the cell-centered finite volume method see appendix A.

#### Hydraulic properties (numerical)

In order to numerically calculate the effective intrinsic permeability of the digitized rock samples we calculate the fluxes under creeping flow condition based on an optimized Stokes-solver for cartesian grids. The parallelized Finite Difference-based Stokes-solver is suitable for the calculation of effective hydraulic parameters for low and high-porosity materials^27^. Using volume averaging technique, we coarse-grain the local velocity field ***u***(***x***) obtaining the global velocity component $v$ in flow direction. The intrinsic permeability $k$ is calculated with Darcy’s law according to

5 $$k=\frac{{\eta }_{{\rm{f}}{\rm{l}}}v}{\Delta p},$$

where ${\eta }_{{\rm{f}}{\rm{l}}}$ and $\Delta p$ denote dynamic viscosity of the pore fluid and pressure difference between inflow and the outflow reservoir, respectively. The dynamic viscosity of water ${\eta }_{{\rm{fl}}}$ at 293.15 K is given in Kestin *et al*. (1978) by 1.002 $\times$ 10${}^{-3}$ Pa s^28^.

### Theoretical estimates of laboratory and numerical investigations

#### Analytical prediction of elastic properties for an open-cell structure

Several previous studies suggested that the mechanical behaviour of open-cell can be estimated by relating its structure with the mechanical properties of the material forming the cell walls^29^. One of the most important structural characteristics of foam are relative density and to which extent the cells are open or closed. Open-cell structure can be modelled as a cubic array of members of length $l$ and edge thickness $T$ assuming isotropy^29^. The relative density of the cell, ${\rho }^{\ast }{{\rho }_{{\rm{m}}}}^{-1}$, with ${\rho }_{{\rm{s}}}$ the density of the solid forming the matrix of the foam and ${\rho }^{\ast }$ the density of the foam, is related to the dimensions $l$ and $t$ by

6 $$\frac{{\rho }^{\ast }}{{\rho }_{{\rm{m}}}}\propto {\left(\frac{t}{l}\right)}^{2}.$$

Shear modulus ${\rm{\mu }}$ and the bulk modulus $K$ can be expressed according to

7 $${\rm{\mu }}=\frac{3{E}_{{\rm{s}}}}{8}{\left(\frac{{\rho }^{\ast }}{{\rho }_{{\rm{m}}}}\right)}^{2},\,{\rm{and}}\,$$

8 $$K=\frac{{E}_{{\rm{s}}}{\rho }^{\ast }}{9{\rho }_{{\rm{m}}}},$$

where ${E}_{{\rm{s}}}$ denotes Young’s modulus of the cell wall-forming material. In our estimation, we substitute cell diameter and width of the reticulite structure for length and edge thickness. Cell diameters were obtained by segmentation of the XRCT-images. The micro-CT image was edited by an interactive thresholding method. The gray level image is transformed into a binary image and the relevant information of the raw micro-CT image, in this case corresponding air pixels, are assigned to a specific gray level intervall. In a second step, the large segmented air voxels are separated by computed lines of a watershed, distance transform, and numerical reconstruction algorithms. This results into individual labeled air voxels which are used for further calculations. Young’s modulus ${E}_{{\rm{s}}}$ of the solid frame is calculated from ${v}_{{\rm{P}}}$, ${v}_{{\rm{S}}}$, and ${\rho }_{{\rm{m}}}$ measured by Schilling *et al*.^21^.

#### Estimation of permeability with different theoretical models

Three established theoretical permeability models were chosen to estimate the permeability of reticulte, that cannot be acquired by laboratory experiments. These methods are initially developed for flow through porous media, and are based on mean particle diameter ${d}_{{\rm{p}}}$ and porosity $\phi$. For example, Innocentini *et al*. (1999) and Gunashekar *et al*. (2015) showed that these methods can also be applied to open-cell foams, whose structure is comparable to the one of reticulite^30,31^. The applied models of Kozeny and Carman (KC)^32,33^, Du Plessis and Masliyah (DPM)^34^ and Ergun’s expression (E)^35^ write as

9 $${k}_{{\rm{K}}{\rm{C}}/{\rm{E}}}=\frac{{{d}_{{\rm{p}}}}^{2}{\phi }^{3}}{180/150{(1-\phi )}^{2}},\,{\rm{a}}{\rm{n}}{\rm{d}}\,$$

10 $${k}_{{\rm{DPM}}}=\frac{{{d}_{{\rm{p}}}}^{2}\phi \left[1-{\left(1-\phi \right)}^{0.33}\right]\left[1-{\left(1-\phi \right)}^{0.66}\right]}{63\ {(1-\phi )}^{1.33}},$$

respectively. In comparison to the structure of foam, the one of reticulite does not consist of repetitive cells and thus, cell diameter is used rather than particle diameter. The relation between particle diameter ${d}_{{\rm{p}}}$ and cell diameter ${d}_{{\rm{c}}}$^30,36,37^ writes as

11 $${d}_{{\rm{p}}}=\frac{1.5{d}_{{\rm{c}}}\left(1-\phi \right)}{\phi }.$$

## Results

### Microstructure

**Figure 2:**
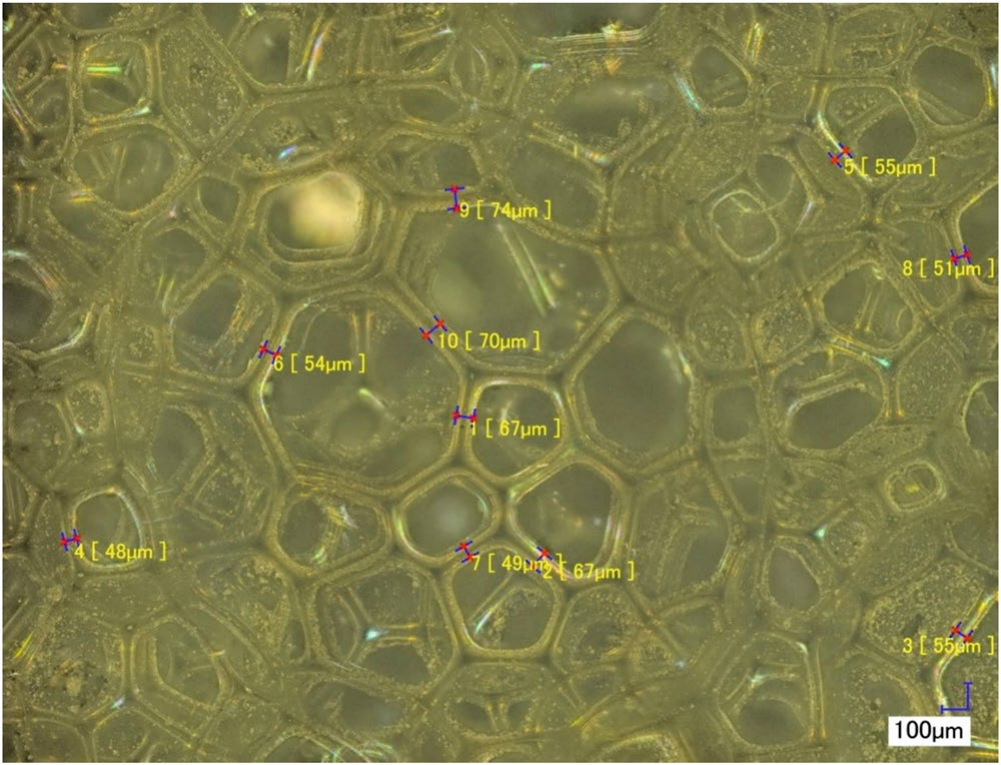
Surface view of the reticulite sample by combination of scans at 200x magnification. Ten measurements were performed to estimate “strut” diameter using the VHX-2000 communication software. The averaged “strut” diameter on ten measurements for 200x magnification is around 59 $\mu$m.

**Figure 3:**
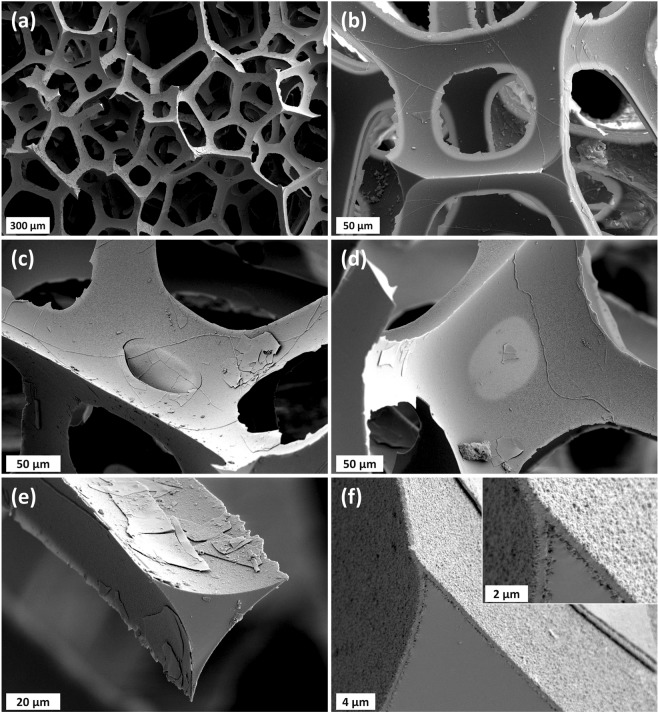
Scanning electrone microscope (SEM) images of reticulite showing (**a**) the general framework of “struts”, (**b**) a four-sided comb with broken and removed skin, (**c**) a comb with partly broken skin, (**d**) a comb with intact skin, (**e**) a cross-section of a strut with fragments, and (**f**) the internal structure of a “strut”.

**Figure 4:**
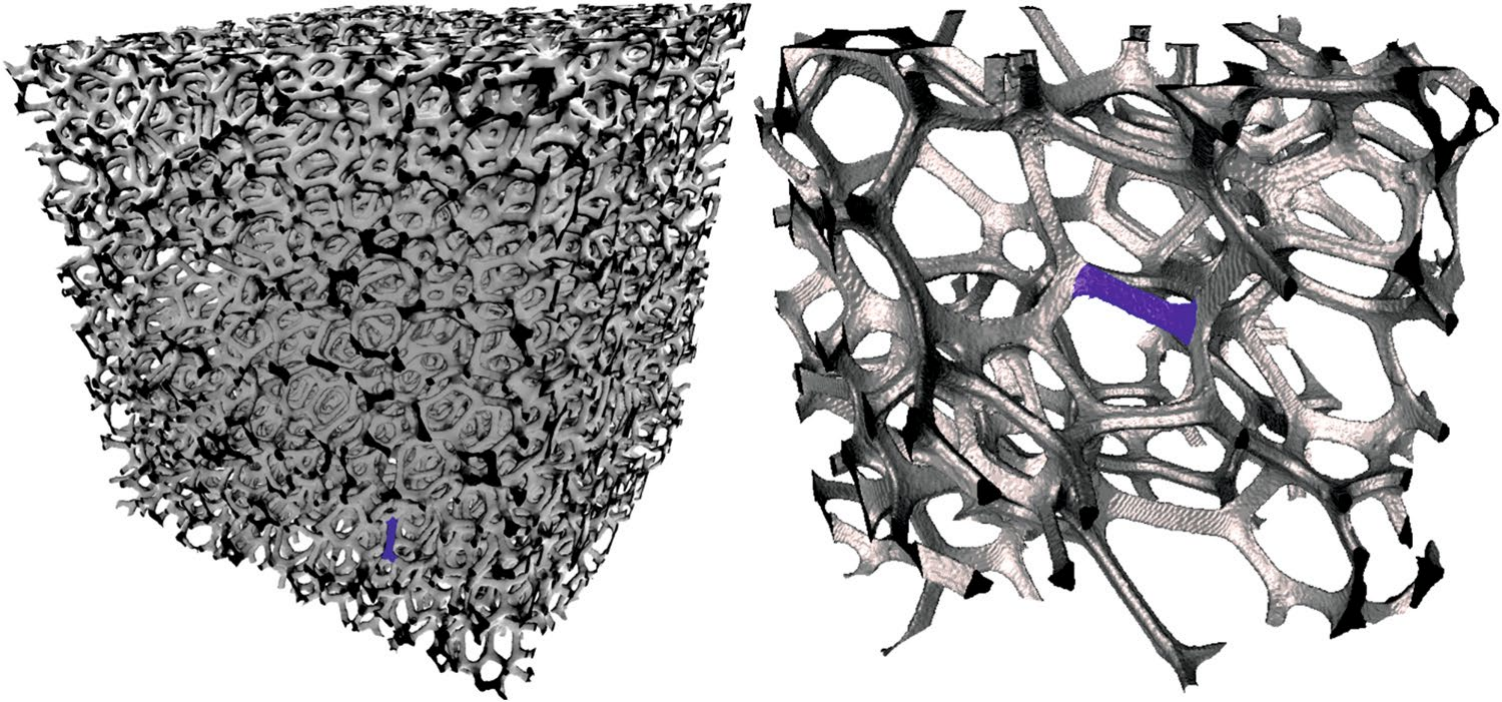
Segmented images of the low- (left) and high-resolution (right) XRCT-scans of the two dry reticulite samples (segmented-subsamples.rar in Schepp *et al*.)^17^. One single strut is marked in blue in both plots for orientation.

**Figure 5:**
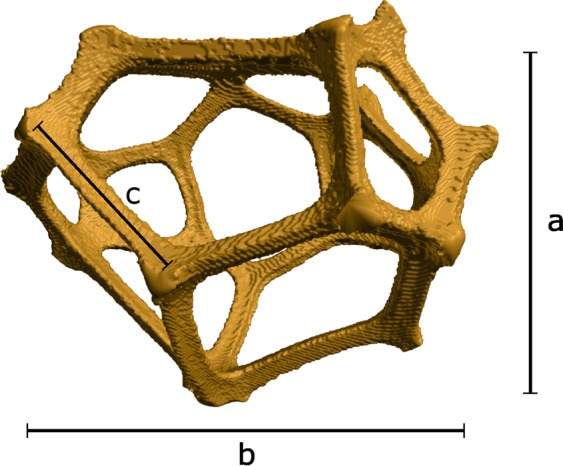
Typical form of an elementary cell of reticulite extracted from the high-resolution XRCT-image. Here, the lengths $a$, $b$, and $c$ estimate to 904 $\mu$m, 1178 $\mu$m, and 318 $\mu$m, respectively.

**Figure 6:**
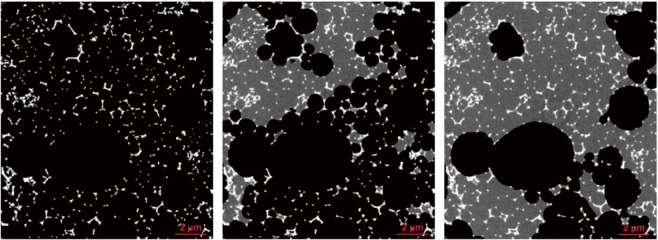
2D snapshot images (left to right) from time-lapse XRCT-scans monitoring the saturation process of reticulite with distilled water. White, gray and black color indicate skeleton of reticulite, water saturated and air-filled pore space. The area of each image corresponds to 800 $\times$ 800 voxels with an edge length of 12.85 $\mu$m.

Reticulite shows an analogy to man-made foams or spongeous bones^29^. The skeleton is built from a regular frame of combs that are formed by struts. The cross sections of these struts have a triangular shape with a thickness ranging between approximately 45 $\mu$m and 75 $\mu$m as deduced from digital microscopy (Fig. 2) and SEM measurements (Fig. 3). The network of struts forms quadrilateral, pentagonal or hexagonal combs that are arranged around spherical void structures of different size (Figs. 3a, 4, 5, 6).

Surface measurements performed with a digital microscope suggest that approximately 30 % of the combs in the investigated sample possess an intact skin (Fig. 2). In contrast, SEM images on a different sample only sporadically show intact skins that are limited to comparatively small quadrilateral combs (Fig. 3). On the one hand, it seems reasonable to assume that, in general, skins at a sample’s outer face are more likely to be destroyed even by careful sample treatment than skins on the inside that were invisible to our non-invasive imaging technologies. On the other hand, our XRCT measurements (in principle capable of visualizing the internal structure) did not show any skin connecting the combs, but the resolutions of our XRCT measurements fell short of the thickness of the skins of less than 1 $\mu$m (Fig. 4).

The solid skeleton is often covered by platy fragments that seem to adhere to the strut surface (Fig. 3c,e). These fragments show a striking similarity to the fracture patterns of skin fragments at strut flanks (Fig. 3b). It seems likely that these fragments originate from former skins.

The structure of the struts shows a clear separation into two components (Fig. 3f): (1) a shell with a thickness of approximately 0.5 $\mu$m with a homogeneous inner layer and a granular outer structure on both sides, i.e., towards the inside and outside of the strut, with a maximum grain size of about 0.2 $\mu$m, and (2) a highly homogeneous, amorphous inner material. An EDS spectrum of the latter revealed 30 % C, 21 % Si, 15 % Fe, 13 % O, 9 % Ca and minor constituents of Al, Mg and Ti.

Based on the results of our microstructural investigations we conclude that the majority of struts are not connected by skins and therefore, permeability is not controlled by skinned combs forming signfifcant amounts of dead ends or volumes. To further confirm our conclusions on the connectivity of reticulite combs we saturated one sample with distilled water using a pipette in accordance with the laboratory saturation experiment described earlier, and documented the saturation progress by XRCT-measurements. Following a time delay of 5 min the XRCT-scan started with a duration of 3 h. The fluid movement was observed during scanning resulting in a diffuse water-phase in the grey-value images. Visual inspection of the time-lapse images (Fig. 6) confirm that (1) reticulite combs are connected, and (2) that water has a higher affinity to wet the bulk surface of reticulite than air.

In contrast to reservoir rocks^38^, it was technically straightforward to digitize the structure of reticulite with XRCT measurements (Figs. 4 and 5) by segmenting the image-enhanced datasets of the dry sample into two classes (mineral and pore) using global thresholds for the covered range of grey-values. The low-resolution image (Fig. 4, left hand side) was used to for numerical modelling of effective material properties.

### Basic physical properties

The matrix density ${\rho }_{{\rm{m}}}$ was determined for 7.3 g of reticulite sample powder in a 25 ml pycnometer and amounts to (2785 $\pm$ 1) $\,{\rm{kg}}{{\rm{m}}}^{-3}$. The average geometric density for two cylindrical samples varying in dimension was calculated as (46 $\pm$ 5) $\,{\rm{kg}}\,\ {{\rm{m}}}^{-3}$ with a standard deviation of 1 kg m${}^{-3}$. The resulting total porosity ${\phi }_{{\rm{tot}}}$ amounts to (98.3 $\pm$ 0.2) %.

### Elastic properties

#### laboratory

Determining the ultrasound velocity of reticulite is challenging from a laboratory point of view. The loss of signal intensity even along sample dimensions (and travel paths) as small as 1 cm is significant. For a 400 V source signal with 1 MHz central frequency no robust first arrival could be identified exceeding the reduced noise level after stacking of 1000 traces. The amplitude of the transmitted signal increased with decreasing frequency. We were technically limited to a minimum frequency of 100 kHz. The maximum ultrasound P-wave velocity ${v}_{{\rm{P}}}$ was determined for the highest signal-to-noise ratio at 100 kHz signal frequency for 100 kHz transducers and amounts to (2561 $\pm$ 244) $\,{\rm{m}}{{\rm{s}}}^{-1}$. S-wave first arrivals could not be identified for the applied test conditions.

#### Numerical

The results of the two-phase wave-propagation simulations to estimate effective elastic properties for the dry and the water-saturated case are P-wave and S-wave velocities, bulk modulus and shear modulus of ${v}_{{\rm{P,dry}}}\ =$ 1995 ms${}^{-1}$ and ${v}_{{\rm{P,sat}}}\ =$ 1852 ms${}^{-1}$, ${v}_{{\rm{S,dry}}}\ =$ 954 ms${}^{-1}$ and ${v}_{{\rm{S,sat}}}\ =$ 778 ms${}^{-1}$, ${K}_{{\rm{dry}}}\ =\ 0.743$ GPa and ${K}_{{\rm{sat}}}\,=$ 3.057 GPa and ${{\rm{\mu }}}_{{\rm{dry}}}=$ 0.244 GPa and ${{\rm{\mu }}}_{{\rm{sat}}}=$ 0.707 GPa. Additionally all results are summarized in Table 2 for a direct comparison with measured and theoretical estimated results. Because of the limited contact points for the high-resolution image to the homogeneous embedding in the numerical setup we concentrate on the low-resolution image. For these time-of-flight simulations we consider the high-frequency limit of the Biot approach for the fluid saturated cases as described in section 2.4.1.

**Table 2:** Overview of all volume properties, transport properties, and elastic moduli as determined by laboratory measurements, numerical estimations, and theoretical estimations for reticulite samples. Numerical and theoretical estimations were performed for indicated low- and high-resolution XRCT-scans (see text for discussion).

	parameter	laboratory	numerical	theoretical
		measurement	estimation	estimation
volume property	${\rho }_{{\rm{m}}}$ (kg m${}^{-3}$)	2785 $\pm$ 1	—	—
	${\rho }_{{\rm{geo}}}$ (kg m${}^{-3}$)	46 $\pm$ 5	—	—
	${\phi }_{{\rm{tot}}}$ (%)	98.3 $\pm$ 0.2	$\blacktriangle$ 93.05	—
			▽ 88.05	
	${\alpha }_{{v}_{rmP,{\rm{sat}}}}$ (—)	—	▽ 1.155	—
	${\alpha }_{{v}_{{\rm{P}},{\rm{sat}}\ast }}$ (—)	—	▽ 1.185	—
	${\alpha }_{{v}_{{\rm{S}},{\rm{sat}}}}$ (—)	—	▽ 1.187	—
	${\alpha }_{{v}_{{\rm{S}},{\rm{sat}}\ast }}$ (—)	—	▽ 1.182	—
transport property	${v}_{{\rm{P}},{\rm{dry}}}$ (m s${}^{-1}$)	2561 $\pm$ 244	▽ 1995	—
	${v}_{{\rm{P}},{\rm{sat}}}$ (m s${}^{-1}$)	—	▽ 1852	—
	${v}_{{\rm{P}},{\rm{sat}}\ast }$ (m s${}^{-1}$)	—	▽ 1667	—
	${v}_{{\rm{S}},{\rm{dry}}}$ (m s${}^{-1}$)	—	▽ 954	—
	${v}_{{\rm{S}},{\rm{sat}}}$ (m s${}^{-1}$)	—	▽ 778	—
	${v}_{{\rm{S}},{\rm{sat}}\ast }$ (m s${}^{-1}$)	—	▽ 330	—
	$\lambda$ (W m${}^{-1}$ k${}^{-1}$)	0.709	$\blacktriangle$ 0.269	—
			▽ 0.703	
	$k$ (m${}^{2}$)	—	$\blacktriangle$ 5.54 $\times$ 10${}^{-9}$	3.18 $\times$ 10${}^{-9}$ (after^35^)
				2.65 $\times$ 10${}^{-9}$ (after^32,33^)
				7.25 $\times$ 10${}^{-9}$ (after^34^)
			▽ 5.61 $\times$ 10${}^{-9}$	6.88 $\times$ 10${}^{-9}$ (after^35^)
				5.73 $\times$ 10${}^{-9}$ (after^32,33^)
				1.97 $\times$ 10${}^{-9}$ (after^34^)
**elastic modulus**	${K}_{{\rm{dry}}}$ (GPa)	—	—	$\blacktriangle$ 1.608 wm
				$\blacktriangle$ 0.190 mf
			▽ 0.743	▽ 0.228 wm
				▽ 0.117 mf
	${K}_{{\rm{sat}}}$ (GPa)	—	▽ 3.057	—
	${K}_{{\rm{sat\ast }}}$ (GPa)	—	▽ 36.29	—
	${{\rm{\mu }}}_{{\rm{dry}}}$ (GPa)	—	—	$\blacktriangle$ 6.600 wm
				$\blacktriangle$ 0.011 mf
			▽ 0.244	▽ 1.932 wm
				▽ 0.004 mf
	${{\rm{\mu }}}_{{\rm{sat}}}$ (GPa)	—	▽ 0.707	—
	${{\rm{\mu }}}_{{\rm{sat\ast }}}$ (GPa)	—	▽ 1.5	—

${\rho }_{{\rm{m}}}$: matrix density; ${\rho }_{{\rm{geo}}}$: geometrical density; ${\phi }_{{\rm{tot}}}$: total porosity; $\alpha$: tortuosity; ${v}_{{\rm{P}}}$: P-wave velocity; ${v}_{{\rm{S}}}$: S-wave velocity; $\lambda$: thermal conductivity; $k$: permeability; $K$: bulk modulus; ${\rm{\mu }}$: shear modulus; ▽: low-resolution XRCT-scan; $\blacktriangle$: high-resolution XRCT-scan; dry, sat, sat ${}^{\ast }$: air-, water-, and high-density fluid-filled pores; wm: weighted mean; mf: most frequent.

**Figure 7:**
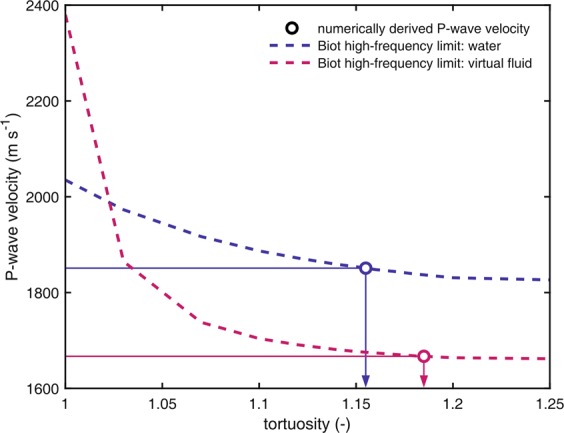
P-wave velocity of the high-frequency limit of the Biot approach in dependence of the tortuosity using the estimated dry moduli for the two saturation scenarios. With the numerically determined P-wave velocities 1667 m ${s}^{-1}$ and 1851 m s${}^{-1}$ we can estimate the free parameter tortuosity for water and high-density fluid saturation, respectively.

The tortuosity can be estimated when the dry and saturated moduli are known. We consider the case for a saturation with water and with a virtual high-density fluid (cf. Table 2). By using equation (1) it is straightforward to estimate the tortuosity using the shear moduli. We obtain the tortuosities of ${\alpha }_{{{\rm{v}}}_{{\rm{S,sat}}}}=1.187$ and ${\alpha }_{{{\rm{v}}}_{{\rm{S,sat}}}^{\ast }}=1.182$ for water and high-density fluid saturation, respectively. Inverting ${v}_{{\rm{p}}}$ for the high-frequency limit to tortuosity is not straightforward. Therefore, we calculate the theoretical value of the P-wave velocity for the saturated cases in dependence of the dry moduli (Table 2) and the tortuosity using the Biot fomulas^8^. By comparing the numerical values for the P-wave velocities for the saturated cases with the ones in dependence of the tortuosity (Fig. 7) it is possible to obtain ${\alpha }_{{{\rm{v}}}_{{\rm{P,sat}}}}=1.155$ and ${\alpha }_{{{\rm{v}}}_{{\rm{P,sat}}}^{\ast }}=1.185$ for water and high-density fluid saturation, respectively.

#### theoretical estimates

**Figure 8:**
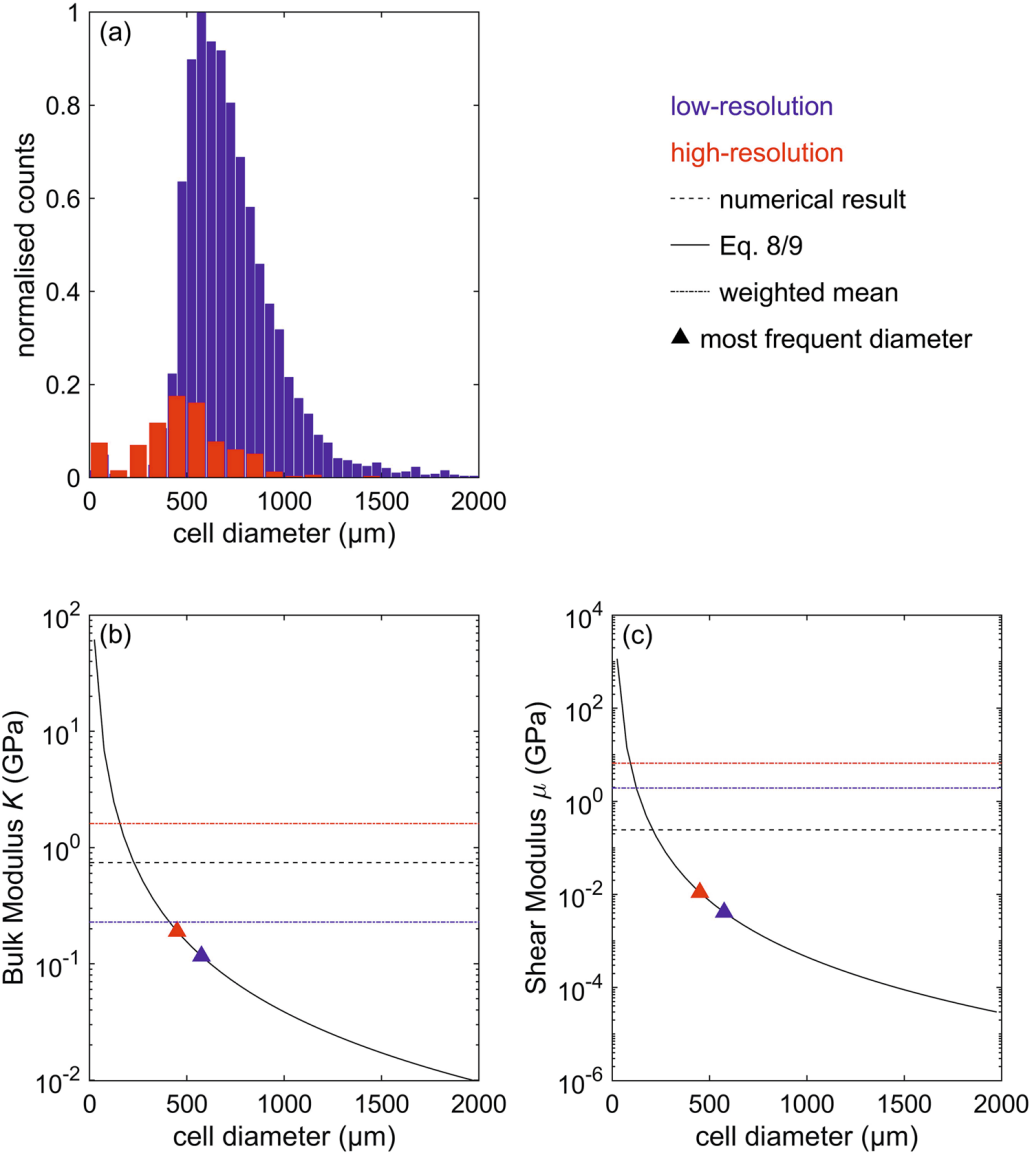
(**a**) Cell-diameter distribution, (**b**) theoretically calculated bulk modulus, and (**c**) shear modulus as derived from low- (blue) and high-resolution XRCT-scans (red). In (**b,c**), the dotted and solid black lines correspond to the numerical and the theoretical estimations of bulk and shear modulus, respectively. Triangles represent bulk and shear modulus of the predominant cell diameters of the scanned samples.

The calculated distributions of cell diameter –as extracted from the different $\mu$CT-scans– exhibit no homogeneous pattern (Fig. 8a). The mean edge thickness amounts to 59 $\mu$m. Bulk modulus $K$ and shear modulus ${\rm{\mu }}$ were theoretically calculated based on the predominant cell diameters of the low- and high-resolution histograms (575 $\mu$m and 450 $\mu$m) and for the weighted mean of all cell diameters (722 $\mu$m and 478 $\mu$m) (Fig. 8b,c). Furthermore, elastic moduli where predicted for every cell diameters up to 2000 $\mu$m (Fig. 8b,c). Theoretically calculated bulk and shear moduli for low-resolution $\mu$CT-scan are ${K}_{{\rm{dry}}}$= 0.228 and 0.117 GPa and ${{\rm{\mu }}}_{{\rm{dry}}}$= 1.932 and 0.004 GPa for weighted mean cell diameter and predominant cell diameters. The moduli are in the same order of magnitude than the numerically determined ones (Table 2) or up to two magnitudes higher.

### Thermal properties

#### Laboratory

The thermal conductivity of reticulite was too low to correctly apply the method of Popov (1997) that relies on comparing the relative change in temperature after heating the sample with that of standards with known properties^18^. Yet, the upper limit for the thermal conductivity can be set to 0.709 W m${}^{-1}$ K${}^{-1}$. However, the thermal conductivity is probably much lower, due to the high amount of air with a thermal conductivity of 0.0262 W m${}^{-1}$ K${}^{-1}$^39^.

#### Numerical

**Figure 9:**
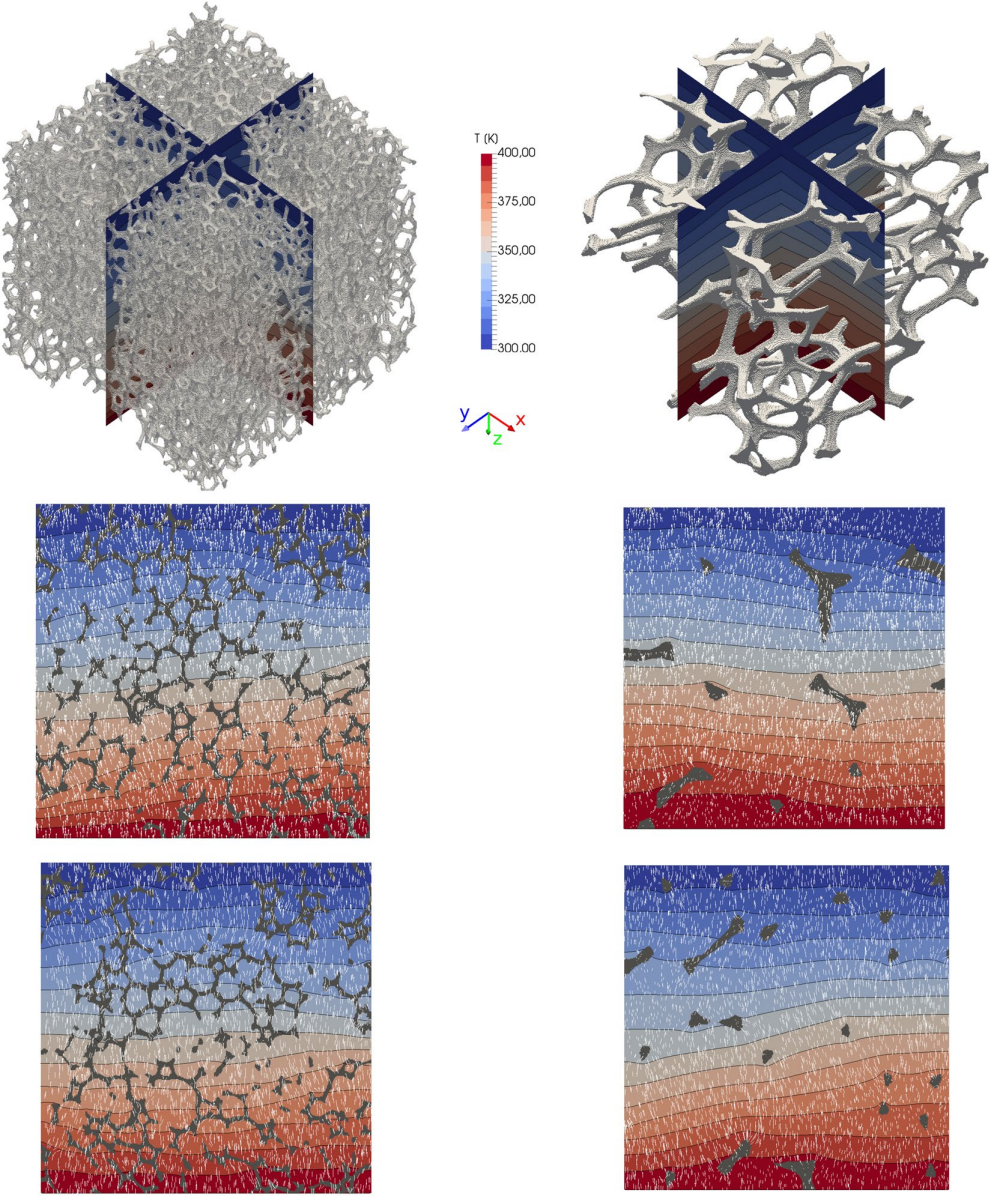
Numerically derived temperature distribution and gradient vectors within the low- (left) and high-resolution XRCT-images. On top, the orientation of representative 2D sections in $xz$- (middle) and $yz$-plane (bottom) are shown in relation to the 3D volume. The solid matrix of reticulite is indicated by grey colours. Temperature isolines are indicated by black lines. White arrows represent temperature gradient vectors.

**Figure 10:**
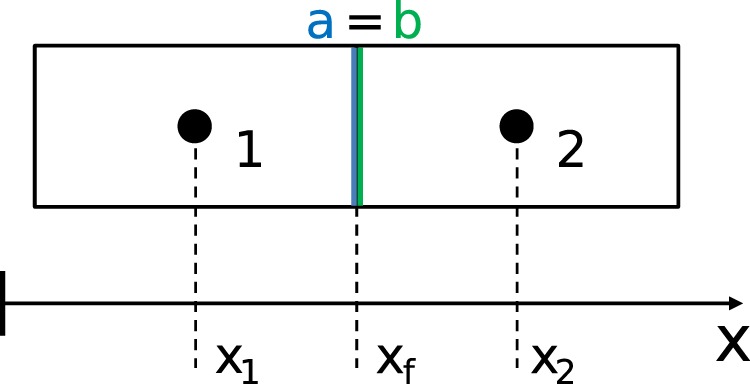
Schematic representation of two adjacent cells 1 and 2 in 1D. Cells are connected by faces $a$ and $b$ at ${x}_{{\rm{f}}}$.

The effective thermal conductivities of the low- and high-resolution XRCT-scans estimate to ${\lambda }_{{\rm{e}}{\rm{f}}{\rm{f}}}\,=$ 0.703 W m${}^{-1}$ K${}^{-1}$ and ${\lambda }_{{\rm{e}}{\rm{f}}{\rm{f}}}\,=$ 0.269 W m${}^{-1}$ K${}^{-1}$, respectively. The variation between both results can largely be explained by the difference in natural material variability. The rock volume fraction of the low-resolution scan is approximately two times larger than the one of the high-resolution scan due to the difference in determined total porosity. An overview of the numerically determined spatial temperature distribution and vector field is given in Figs. 9,10.

### Hydraulic properties

#### Laboratory

Pores in investigated reticulite samples are interconnected. Samples were tested with distilled water and silicon oil. Notably, distilled water was absorbed by the sample and distributes across complicated fluid pathways. Because the potentially high capillary forces and the related risk of damaging hydraulically relevant skins just by fluid movement, silicon oil was applied. The inflow of silicon oil occurred more slowly than the inflow observed for distilled water, and the full amount of oil was ingested by the sample after several hours.

#### Numerical

In addition to laboratory measurements, total porosity and permeability were also estimated from segmented XRCT-images (Table 2). In our numerical simulations the differential pressure $\Delta$p is 1.4 $\times$ 10${}^{-4}$ Pa m${}^{-1}$ and 5.1 $\times$ 10${}^{-4}$ Pa m${}^{-1}$ for the low- and high-resolution image, respectively. The fluid viscosity ${\eta }_{{\rm{fl}}}$ amounts to 1.2 Pa s. The numerically derived permeabilities for the low- and high-resolution images are in fairly good agreement (Table 2), that is, 5.62 $\times$ 10${}^{-9}$ m${}^{2}$ compared to 5.54 $\times$ 10${}^{-9}$ m${}^{2}$, respectively.

With the calculated permeability and the estimated porosity of reticulite the reference frequency of the Biot theory can be estimated for the saturated case with the equation described above. The resulting reference frequency amounts to about 25 Hz. Such a low Biot frequency is in contrast to the majority of rocks investigated whose Biot frequency is typically in the MHz-range^8^. However, on the other hand such a low Biot reference frequence was also reported for other materials like cancellous bones^40^.

#### Theoretical estimates

The permeability of reticulite was estimated from two independent structural properties, that is, mean cell diameter ${d}_{{\rm{p}}}$ = 711 $\mu$m and total porosity (0.9305 for high-resolution and 0.8805 for low-resolution XRCT-scan), according to three different theoretical models (equations (9) to (11)). Both structural properties were derived from the low- and high-resolution XRCT-scans (Table 2). The theoretically calculated permeabilities are between $k=2.65\ \times \ 1{0}^{-9}$ m${}^{2}$ and k = 7.25 $\times$ 10${}^{-9}$ m${}^{2}$ for the high-resolution XRCT-scan and between k = 1.97 $\times$ 10${}^{-9}$ m${}^{2}$ and k = 6.88 $\times$ 10${}^{-9}$ m${}^{2}$ for the low-resolution XRCT-scan. That means they differ by up to half an order of magnitude among each other, but are in the same order of magnitude than the numerically determined ones (Table 2).

## Discussion

Reticulite is a very fragile rock with an exceptionally high porosity. It can be regarded as an end-member, because the ratio between pores and solid is vice versa in comparison to standard reservoir rocks and most man-made technical materials. The skeleton of reticulite can be evaluated and imaged by digital microscopy, X-Ray microtomography, and Scanning Electron Microscope. The geometrical analysis demonstrates the high regularity of the skeleton with an average co-ordination number of four.

Laboratory methods to image the structure of the material are inherently limited by their resolution. For example, the skins partially connecting the network of “struts” were only visible in the microscope and in SEM images. We estimated the thickness of the skins to well below below 1 $\mu$m^7^, i.e., these structures were not adopted for numerical modelling based on the XRCT images potentially causing the remaining difference between laboratory and numerical results on effective elastic properties. Yet, a difference of less than 20 % between laboratory and numerical results considering the experimental uncertainty appears to be a fair agreement.

The porosity derived from laboratory and numerical approaches was in good agreement ($<$ 6% deviation) and could be evaluated with a low uncertainty based on our XRCT images. It was straightforward to distinguish the solid and pore phases during the segmentation workflow based on their grey values, also because reticulite consists of one single mineral phase.

Ultrasonic velocity measurements are challenging because of the high signal attenuation during testing. To ensure an optimal contact to the transducers, the fragile surface of reticulite had to be prepared with caution. Therefore, we restricted ourselves to the determination of P-wave velocities for the dry case, which required high experimental efforts. Saturating the sample led to externally visible damage, so it can be assumed that the skins/structure inside is probably also damaged. P-wave velocities from numerical calculation of elastic properties were lower than the measured ones also exceeding the experimental uncertainty. In addition to the unknown contribution of the skins we see two potential reasons for this deviation: (1) First there is no perfect consistency of the frequency spectrum for both methods. Second, infinitely extended source (periodic boundary conditions) with perfect coupling for the numerical calculation is compared to finite source extension with imperfect coupling for the ultrasonic measurements, and (2) the matrix of reticulite has a structure that is clearly separated into two components (Fig. 3f), in which the outer shell could have properties different to those of the intact lava, whose properties were used for numerical simulations.

It may not be appropriate to assign the full elastic moduli of the mineral measured for fully intact crystal structures to the solid phase of digital images of reticulite. A visual inspection of microscopic images suggested to assign reduced mineral properties because of the visible defects in the struts. This observation is consistent with the findings in Madonna *et al*. (2012), Andrä *et al*. (2013) and Saenger *et al*.^3,38,41^. Please note that the velocities of the lava we applied to our numerical calculation and theoretical estimates were measured for artificial and inclusion free glasses^21^. Yet, due to the high uncertainties in laboratory velocity measurements we do not want to propose an exact digital rock physics template value as proposed in Saenger *et al*.^38^.

Because it was not possible to measure S-waves for the applied test conditions we could not calculate the elastic moduli and compare them to numerical results. However, we have predicted elastic moduli for the open-cell structure of the reticulite (see section 2.5.1). The large deviation of these elastic moduli for every single cell diameter (Fig. 8, solid line) and most frequent cell diameter (Fig. 8, rectangle) and the numerical results (Fig. 8, dashed line) for dry case low-resolution image (see Table 2) could be explained by the fact that, in contrast to numerical approaches, predictions do not take into account the size distribution of the cell diameters. The moduli of the weighted mean (Fig. 8, dashed pointed line) account for the size distribution but there is a gap in the histogram at lower diameters which plays no role in our numerical calculations.

The tortuosity can also be evaluated very accurately with the proposed workflow. The two saturation scenarios provided consistent estimates for the P-wave as well as for the S-wave case representing one important result of this study using such a high-porosity volcanic rock. Especially the simulated very slow S-wave velocities for the case where we saturated with the virtual fluid with a high density were remarkable from a numerical point of view, though expected.

The measured thermal conductivity was consistent with the numerical results (Table 2), the results of the low- and high-resolution scans remained below the laboratory measurement, which is regarded as the upper limit for thermal conductivity of reticulite.

Because of the unresolved skins in the digital images the values of the permeability have to be regarded as an upper bound. However, due to their fragility they may not stand any fluid flow. An indication is the full saturation of all small elementary cells in our time-lapse experiment (see Fig. 6, right hand side): No isolated unsaturated small elementary cells are visible.

A direct comparison of laboratory and digital rock physics remains difficult because of the highly challenging sample preparation for the corresponding measurements. However, we found no severe contradiction when comparing results of the different and therefore complementing methods. Furthermore, estimates of permeability from theoretical models using two properties of regular reticulite structure were consistent in magnitude with the numerical results (Table 2).

Although laboratory methods should generally be favoured over numerical and theoretical approaches, the illustrated DRP workflow is the most appropriate way to describe the studied material considering the overall “cost-and-benefit” analysis. Laboratory measurements were generally very challenging to realize and required enormous effort for a moderate outcome. Porosity, effective elastic properties, thermal conductivity and permeability of reticulite could be determined adequately or at least in fair agreement to experimental results. Also the theoretical estimations provided useful results, which was not necessarily expected, since the structure of reticulite does not comply with the assumptions behind these estimates. Depending on the requirements of the desired application, numerical methods as well as theoretical estimates may be sufficient and appropriate for the characterization of such a highly porous material.

## Conclusions

Reticulite samples from Hawaii are characterized with traditional experimental approaches such as microscopy, ultrasonic velocity measurements, and a digital rock physics workflow. It complements the considerations of May *et al*. (2015) and Mangan and Cashman (1996)^6,7^. Values for the porosity, elastic properties, tortuosity, permeability and thermal conductivity were presented and discussed. We demonstrated that the digital rock physics workflow yields reasonable results for high-porosity rocks. Especially, the tortuosity values consistently determined for different saturation scenarios showed that we could determine the high- and low-frequency velocities for the Biot-approach. Our considerations on reticulite samples suggests to use reduced mineral moduli (compared to the moduli of fully intact minerals) for identified mineral phases to estimate effective elastic properties using high-resolution XRCT-scans of rocks. Depending on the fields of application, numerical methods as well as theoretical estimates can become valid alternatives to laboratory methods for highly porous materials like reticulite.

## Data Availability

The datasets generated during and/or analysed during the current study are available on DaRUS (The data repository of the University of Stuttgart), cf [10.18419/darus-680] and on the ROCKETH webpage [https://rockphysics.org/index.php/downloads].

## Appendix

The approach in Eq. (4) results in one algebraic equation for each cell $P$. These equations write as ${a}_{P}\ {T}_{P}+{\sum }_{nb}{a}_{{\rm{k}}}\ {T}_{{\rm{k}}}={b}_{{\rm{P}}}$, where index $nb$ denotes the number of neighboring cells connected with cell $P$ based on its discretization stencil. All these equations are combined in a matrix equation that gives $\overrightarrow{A}\cdot \overrightarrow{T}=\overrightarrow{b}$, where the number of unknowns $\overrightarrow{T}$ is equal to the total number of cells. In our case (i.e. 64 million cells) the direct solution approach of the resulting matrix equation $\overrightarrow{T}={\overrightarrow{A}}^{-1}\cdot \overrightarrow{b}$ might be very time consuming and is thus being replaced by an iterative conjugate gradient method solver^42^.

As boundary conditions a fixed temperature difference between two sample end faces is prescribed, the remaining sides are treated as adiabatic walls. Heat flux is calculated from the temperature distribution across the sample. Furthermore, heat flux is determined at both temperature sides, that is, sample end faces. On the lower side the heat flux is evaluated with forward differences, on the upper side the heat flux is evaluated with backward differences. According to the boundary conditions, effective thermal conductivity can be calculated as follows

$${\lambda }_{{\rm{e}}{\rm{f}}{\rm{f}}}=\frac{\dot{Q}\Delta x}{\Delta TA}=\frac{\dot{Q}}{\Delta T\Delta {x}^{3}},$$

where $$\Delta {x}^{3}$$, $$\Delta $$$$T$$, and $$\dot{Q}$$ denote sample dimension, temperature difference, and heat flux, respectively.

For every XRCT-image a control volume is created and the corresponding thermal conductivity is assigned. The thermal conductivity of air is assumed to be 0.0262 W m${}^{-1}$ K${}^{-1}$^39^. The thermal conductivity of reticulite was estimated by the weighted average of the thermal conductivities of the composing elements and amounts to 18.87 W m${}^{-1}$ K${}^{-1}$. We assumed a simplified fourphase material (cf. section 2.2) composed of SiO${}_{2}$, AlO${}_{3}$, MgO, and CaO (thermal conductivies of minerals according to Čermák und Rybach^43^. The computational mesh consists of 400 $\times$ 400 $\times$ 400 cells and can be viewed as “numerically perfect” (e.g. structured mesh with uniform cube cells). The numerical approach was implemented in the open-source finite volume toolbox OpenFOAM. Two simulation convergence criteria were specified: (1) equal heat fluxes at both temperature sides and (2) heat fluxes do not vary within 10 digits. All simulations were performed in parallel on a 224-core cluster. On average a single calculation took less than three minutes.

## References

[1] Cnudde V, Boone M (2013). High-resolution X-Ray computed tomography in geosciences: A review of the current technology and applications. Earth-Science Reviews.

[2] Fusseis F, Xiao X, Schrank C, Carlo FD (2014). A brief guide to synchrotron radiation-based microtomography in (structural) geology and rock mechanics. Journal of Structural Geology.

[3] Andrä H (2013). Digital rock physics benchmarks - part II: Computing effective properties. Computers and Geosciences.

[4] Dvorkin J, Derzhi N, Diaz E, Fang Q (2011). Relevance of computational rock physics. Geophysics.

[5] Swanson DA, Rose TR, Fiske RS, McGeehin JP (2012). Keanakāko-i tephra produced by 300 years of explosive eruptions following collapse of Kīlauea’s Caldera in about 1500 CE. Journal of Volcanology and Geothermal Research.

[6] May, M., J. Carey, R., A. Swanson, D. & F. Houghton, B. *Reticulite-Producing Fountains From Ring Fractures in Kīlauea Caldera ca. 1500 CE* (Geophysical Monograph Series, 2015).

[7] Mangan MT, Cashman KV (1996). The structure of basaltic scoria and reticulite and inferences for vesiculation, foam formation, and fragmentation in lava fountains. Journal of Volcanology and Geothermal Research.

[8] Mavko, G., Mukerji, T. & Dvorkin, J. *The Rock Physics Handbook: Tools for Seismic Analysis of Porous Media* (Cambridge University Press, 2009), 2 edn.

[9] Macdonald, G. A. & Hubbard, D. H. *Volcanoes of the national parks in Hawaii* (Hawaii Natural History Association, 1974).

[10] Jackson, M. G., Weis, D. & Huang, S. Major element variations in Hawaiian shield lavas: Source features and perspectives from global ocean island basalt (OIB) systematics. *Geochemistry, Geophysics, Geosystems* **13** (2012).

[11] Helz, R., Clague, D., G. Mastin, L. & Rose, T. Electron microprobe analyses of glasses from Kilauea Tephra units, Kilauea Volcano, Hawaii. Tech. Rep., U.S. Geological Survey (2014).

[12] Billiot B, Cointault F, Journaux L, Simon J-C, Gouton P (2013). 3D image acquisition system based on shape from focus technique. Sensors.

[13] Baker DR (2012). An introduction to the application of X-ray microtomography to the three-dimensional study of igneous rocks. Lithos.

[14] Bera B, Mitra SK, Vick D (2011). Understanding the micro structure of Berea sandstone by the simultaneous use of micro-computed tomography (micro-CT) and focused ion beam-scanning electron microscopy (FIB-SEM). Micron.

[15] Landis EN, Keane DT (2010). X-ray microtomography. Materials Characterization.

[16] Voland, V. *et al*. Computed tomography (CT) system for automatic analysis of ice cores. In *10th European Conference on Non-Destructive Testing, ECNDT 2010* (2010).

[17] Schepp, L. L. *et al*. Digital rock physics and laboratory considerations on a high-porosity volcanic rock: micro-XRCT data sets, 10.18419/darus-680, DaRUS, V1 (2020). 10.1038/s41598-020-62741-1 PMC7125207 32246072

[18] Popov YA (1997). Optical scanning technology for nondestructive contactless measurements of thermal conductivity and diffusivity of solid matters. Experimental Heat Transfer, Fluid Mechanics and Thermodynamics.

[19] Saenger EH, Krüger OS, Shapiro SA (2004). Numerical considerations of fluid effects on wave propagation: Influence of the tortuosity. Geophysical Research Letters.

[20] Saenger EH, Gold N, Shapiro S (2000). Modeling the propagation of elastic waves using a modified finite-difference grid. Wave Motion.

[21] Schilling FR, Hauser M, Sinogeikin SV, Bass JD (2001). Compositional dependence of elastic properties and density of glasses in the system anorthite-diopside-forsterite. Contributions to Mineralogy and Petrology.

[22] Gassmann, Über die Elastizität poröser Medien. *Vierteljahrsschrift der Naturforschenden Gesellschaft in Zürich ***96**, 1–23 (1951).

[23] Biot, M. A. Theory of propagation of elastic waves in a fluid-saturated porous solid. i. low-frequency range. *The Journal of the Acoustical Society of America* **28**, 168–178 (1956).

[24] Biot MA (1956). Theory of Propagation of Elastic Waves in a Fluid-Saturated Porous Solid. II. Higher Frequency Range. The Journal of the Acoustical Society of America.

[25] Wang Z (2000). The Gassmann equation revisited: Comparing laboratory data with Gassmannas predictions. Seismic and Acoustic Velocities in Reservoir Rocks.

[26] Patankar, S. V. *Numerical heat transfer and fluid flow*. Series on Computational Methods in Mechanics and Thermal Science (Hemisphere Publishing Corporation (CRC Press, Taylor & Francis Group), 1980).

[27] Osorno M, Uribe D, Ruiz OE, Steeb H (2015). Finite difference calculations of permeability in large domains in a wide porosity range. Archive of Applied Mechanics.

[28] Kestin J, Sokolov M, Wakeham WA (1978). Viscosity of liquid water in the range -8 °C to 150 °C. Journal of Physical and Chemical Reference Data.

[29] Gibson, L. J. & Ashby, M. F. *Cellular Solids: Structure And Properties* (Cambridge University Press, 2014).

[30] Innocentini MD, Salvini VAR, Macedo A, Pandolfelli VC (1999). Prediction of ceramic foams permeability using Ergunas equation. Materials Research.

[31] Gunashekar S, Pillai KM, Church BC, Abu-Zahra NH (2015). Liquid flow in polyurethane foams for filtration applications: a study on their characterization and permeability estimation. Journal of Porous Materials.

[32] Kozeny, J. Über Kapillare Leitung des Wassers im Boden (Aufstieg, Versickerung, und Anwendung auf die Bewässerung). *Denkschriften der Kaiserlichen Akademie der Wissenschaften / Mathematisch-Naturwissenschaftliche Klasse* **136**, 271–306 (1927).

[33] Carman P (1997). Fluid flow through granular beds. Chemical Engineering Research and Design.

[34] DuPlessis JP, Masliyah JH (1991). Flow through isotropic granular porous media. Transport in Porous Media.

[35] Ergun S (1952). Fluid flow through packed columns. Chemical Engineering Progress.

[36] Twigg, M. V. and Richardson, J. T. Preparation and properties of ceramic foam catalyst supports. In *Preparation of Catalysis VI*, vol. 91 of *Studies in Surface Science and Catalysis*, 345–359 (Elsevier, 1995).

[37] Acosta G., F. A., Castillejos E., A. H., Almanza R., J. M. & Flores V., A. Analysis of liquid flow through ceramic porous media used for molten metal filtration. *Metallurgical and Materials Transactions B* **26**, 159–171, 10.1007/BF02648988 (1995).

[38] Saenger EH (2016). Analysis of high-resolution X-ray computed tomography images of Bentheim sandstone under elevated confining pressures. Geophysical Prospecting.

[39] Stephan K, Laesecke A (1985). The thermal conductivity of fluid air. Journal of Physical and Chemical Reference Data.

[40] Steeb H (2010). Ultrasound propagation in cancellous bone. Archive of Applied Mechanics.

[41] Madonna C, Almqvist BS, Saenger EH (2012). Digital rock physics: numerical prediction of pressure-dependent ultrasonic velocities using micro-CT imaging. Geophysical Journal International.

[42] Hestenes, M. R. & Stiefel, E. Methods of conjugate gradients for solving linear systems. *Journal of research of the National Bureau of Standards* **49** (1952).

[43] Čermák, V. & Rybach, L. Thermal conductivity and specific heat of minerals and rocks. In Beblo, M. (ed.) *Geophysics - Physical Properties of Rocks*, chap.Landolt-Bornstein Numerical Data and Functional Relationships in Science and Technology, 305–343 (Springer, 1982).

